# Erythropoietin Pathway: A Potential Target for the Treatment of Depression

**DOI:** 10.3390/ijms17050677

**Published:** 2016-05-06

**Authors:** Chongyang Ma, Fafeng Cheng, Xueqian Wang, Changming Zhai, Wenchao Yue, Yajun Lian, Qingguo Wang

**Affiliations:** School of Basic Medical Sciences, Beijing University of Chinese Medicine, School of Basic Medical Sciences, 11 Beisanhuandong Road, Chao Yang District, Beijing 100029, China; machongyang@live.com (C.M.); fafengcheng@gmail.com (F.C.); shirlyding@163.com (X.W.); zhaichangming1989@163.com (C.Z.); yuewenchao@bucm.edu.cn (W.Y.); niangkuosir@bucm.edu.cn (Y.L.)

**Keywords:** depression, major depressive disorder, erythropoietin, EPO, hippocampal, anti-depressant targets, drug treatment, signaling pathway

## Abstract

During the past decade, accumulating evidence from both clinical and experimental studies has indicated that erythropoietin may have antidepressant effects. In addition to the kidney and liver, many organs have been identified as secretory tissues for erythropoietin, including the brain. Its receptor is expressed in cerebral and spinal cord neurons, the hypothalamus, hippocampus, neocortex, dorsal root ganglia, nerve axons, and Schwann cells. These findings may highlight new functions for erythropoietin, which was originally considered to play a crucial role in the progress of erythroid differentiation. Erythropoietin and its receptor signaling through JAK2 activate multiple downstream signaling pathways including STAT5, PI3K/Akt, NF-κB, and MAPK. These factors may play an important role in inflammation and neuroprogression in the nervous system. This is particularly true for the hippocampus, which is possibly related to learning, memory, neurocognitive deficits and mood alterations. Thus, the influence of erythropoietin on the downstream pathways known to be involved in the treatment of depression makes the erythropoietin-related pathway an attractive target for the development of new therapeutic approaches. Focusing on erythropoietin may help us understand the pathogenic mechanisms of depression and the molecular basis of its treatment.

## 1. Introduction

Depression is the leading cause of psychiatric disability across the globe because of its chronic, treatment-resistant, and recurrent nature; high prevalence; and comorbidity with other chronic neurological and immune diseases [1]. Thus, depression is of major concern in terms of personal happiness and social welfare worldwide. Despite comprehensive biological research, the pathophysiology of depression remains largely unknown. The predominant hypothesis of the underlying mechanism generating depression rests on a low level of brain serotonin (5-hydroxytryptamine, 5-HT) and/or alterations of 5-HT receptors [2] in depressed patients. However, other hypotheses related to neuroinflammation and neuroplasticity are still being considered. For decades, traditional hypotheses of depression have underpinned research into the etiology of depression and *in vitro* testing archetypes; however, many patients continue to suffer from a number of psychiatric syndromes characterized by depressed mood symptoms and a loss of interest. Clinical data indicate that complete symptom remission occurs in only 30%–40% of patients whose treatment with first-line antidepressants is considered successful [3,4]. In addition, most available pharmacological treatment options that target causal factors of depression such as 5-HT and brain-derived neutrophic factor (BDNF) have a significant treatment-onset-response delay and fail to overturn neurocognitive dysfunction [5,6]. Because of these limitations, novel drugs or combinatorial treatments targeting different molecular pathways need to be developed.

In recent years, investigators have started to study inflammation and neuronal plasticity as significant processes underlying depression progression. A growing body of research suggests that depression is an inflammatory [7,8] and neuroprogressive [9,10,11,12] disorder, which could be accompanied by mitochondrial dysfunction [13] and induction of multiple oxidative and nitrosative pathways [14,15]. Both experimental and clinical evidence show that increased concentrations of pro-inflammatory cytokines and glucocorticoids, similar to those in chronically stressful situations and in depression, contribute to the behavioral changes associated with depression [16,17,18]. Targeting these pathways may have the potential to yield antidepressant outcomes.

The hematopoietic growth factor erythropoietin (EPO), known for its role in erythroid differentiation, was first defined as a humoral erythropoietic factor in parabiotic experiments [19] and in anemic plasma preparations [20] in 1950–1955. Since then, accumulating evidence has indicated that EPO has multiple targets and actions [21,22] other than those associated with its erythropoietic effects, similar to many other cytokines and growth factors. It is now widely accepted that EPO not only affects the hematopoietic system, but is also a multifunctional trophic factor that affects the general homoeostasis of the entire organism [23]. EPO has direct effects on immune cells [24], endothelial cells (ECs) [25], and bone marrow stromal cells [26], as well as cells of the heart, gastrointestinal tract, kidney, muscle, reproductive system [27], pancreas [28], and nervous system [29]. EPO is found to be produced in the central nervous system by neurons and astrocytes, where it exerts neurotrophic and neuroprotective effects by binding to EPO receptors (EPOR) in the brain [30,31]. Notably, we now know that in some kinds of acute and chronic neurodegenerative animal models, systemically delivered EPO is able to cross the blood brain barrier (BBB) and exhibits neuroprotective effects and promotion of neuroplasticity [32]. Further, accumulating evidence suggests that EPO has potential antidepressant effects. For this review, we present evidence that EPO-induced signaling pathways are involved in antidepressant activity or regression of depression, and describe the potential of EPO as a novel antidepressant. Ultimately, we hope that these data will lead to the development of EPO and/or its related signaling molecules as adjunct antidepressant therapies.

## 2. Expression of EPO and EPOR in the Nervous System

EPO is a 165-amino-acid protein and a member of the type I cytokine superfamily [33]. Several investigators detected the expression of EPO in other tissues, especially the central nervous system, presenting challenges of the common sense that only the kidney and the fetal liver were able to produce EPO [34]. In addition to the kidney, liver, and uterus, other tissues have been identified as EPO productive and secretory tissues, including peripheral endothelial cells, muscle cells, and insulin-producing cells [35].

Of all the newly identified EPO sites, the presence of EPO in the brain has generated the highest levels of interest and enthusiasm for further investigation. In the brain, the major sites of EPO production and secretion are the hippocampus, internal capsule, cortex, midbrain, cerebral endothelial cells and astrocytes [36,37]. When EPO was first discovered, it was thought that such a large protein could not cross the BBB, and several authors suggested that EPO had a paracrine and autocrine function in some kinds of cells, such as astrocytes [38,39]. However, recent research shows that EPO does indeed have the capacity to cross the BBB, which protects against a variety of potential brain injuries, including transient ischemia and reperfusion [40]. However, in the absence of injury to the BBB, EPO has limited access to the brain [41].

The EPOR, which was first characterized in the 1990s as a polypeptide with a single transmembrane domain and an extracellular domain containing a WSXWS motif [42], is expressed in progenitor cells from hematopoietic cells and ECs, skeletal muscle [43,44,45], and in the brain during the development stage and adulthood [46]. Studies have reported EPOR expression in parts of the nervous system, such as cerebral and spinal cord neurons, hypothalamus, hippocampus, neocortex, dorsal root ganglia, and nerve axons [47,48]. EPORs classically include two EPOR subunits, but may also associate with the β-common receptor (βcR, CD131) subunit [49,50]. This subunit is a crucial part of cytokine receptors such as interleukin (IL)-3, IL-5, and granulocyte-macrophage colony-stimulating factor (GM-CSF) [51], suggesting a potential role of βcR in EPO signaling pathways. Because βcR-knockout mice exhibit normal hematopoiesis [52], it has been suggested that a heteroreceptor complex comprising both EPOR and βcR could, at least partially, mediate the non-hematopoietic functions of EPO [53]. The receptors involved in tissue protection may differ from the hematopoietic EPORs, because some EPO derivatives, such as carbamylated EPO, were shown to mediate neuroprotection without stimulating erythropoiesis [54,55]. Accordingly, it has been suggested that the non-hematopoietic receptor may be a heteromer consisting of one hematopoietic EPOR together with one or more units of the βcR [56,57]. However, other studies detected little overlap of βcR and EPOR expression in the brain [58] and EPO-mediated protection has been demonstrated in cells that do not express βcR at detectable levels [59,60]. Another group of scientists identified ephrin-type B receptor 4 (EphB4) as an EPO receptor that triggers downstream signaling via STAT3 and promotes recombinant human EPO (rhEPO)-induced tumor growth and progression [61]. This receptor has been reported to be not only frequently amplified in some cancers [62], but also over-expressed in the brains of hypoxic-ischemic rats [63]. Through EphB2 signaling, it activates β-catenin *in vitro* and *in vivo* independently of Wnt-signaling and upregulates proneural transcription factors, and thus increases adult hippocampal neurogenesis [64]. Similarly, EphB4 is present and tends to colocalize with EPOR in a subset of cortical neurons [65]. These findings suggest that EPOR may consist of unidentified heterodimeric receptor subunits that may vary between different non-hematopoietic tissues.

In the adult human kidney and fetal liver, the release of EPO into the circulation depends upon tissue oxygen levels and transcription of the *EPO* gene is mediated via hypoxia-inducible factor (HIF)-2α [66]. In most tissues, including the brain, hypoxia-dependent expression of EPO and the EPOR is regulated principally by HIF-1, an a,b-heterodimeric protein which is activated by a collection of stimulators, such as hypoxia [67,68]. Each member of the HIF family, including HIF-1α, HIF-1β, and HIF-3α, appears to play an important role in the regulation of EPO and EPOR expression to protect against hypoxic cell injury [69]. Other cellular disturbances, such as hypoglycemia, increased levels of intracellular Ca^2+^, or intense neuronal depolarization generated by mitochondrial reactive oxygen species (ROS), can increase cerebral EPO expression via HIF activation [70,71,72]. However, the HIF family is not the only factor that can modify the expression of EPO and the EPOR. The GATA transcription factors, key regulators of hematopoiesis, such as GATA1 and GATA2, also contribute to EPO gene regulation [73]. Anemic stress, insulin release and cytokines including insulin-like growth factor, tumor necrosis factor-α [74], interleukin-1β and interleukin-6 [75,76] can also lead to increased expression of EPO and the EPOR in both neuronal and non-neuronal cell populations. Interestingly, a recent study found that hippocampal expression of EPO was decreased in mice by chronic unpredictable stress, and 5-HT could increase EPO expression in the hippocampus [77], which is possible related to verbal and visual learning and memory and spectrum of neurocognitive deficits and mood alterations [78,79]. This finding may highlight EPO as a potential target in the treatment of depression.

## 3. EPO-Induced Intracellular Signaling Pathways

**Figure 1:**
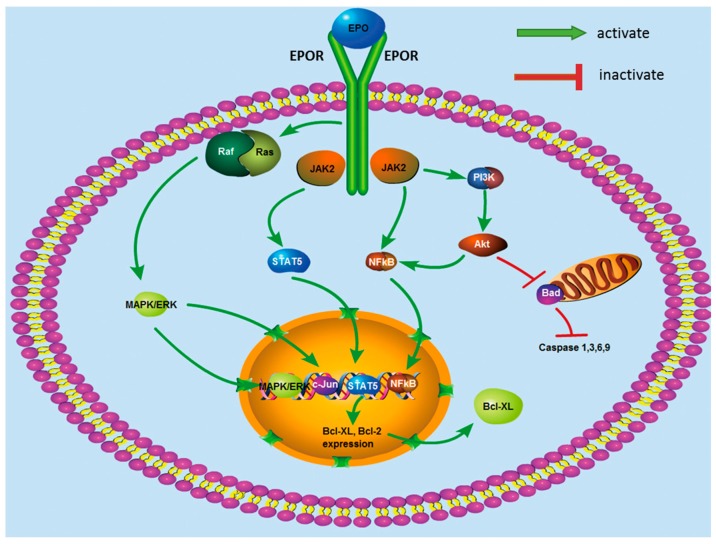
EPO-induced intracellular signaling pathways Erythropoietin and its receptor signaling through JAK2 activate multiple downstream signaling molecules including STAT5, PI3K/Akt, NF-κB, and MAPK. These factors may play an important role in inflammation and neuroprogression in the nervous system.

The existence of the EPO/EPOR signaling pathway has recently been detected in a diversity of systems, but its precise role and function in neurobiology are still controversial (Figure 1). EPO acts via binding to its cell surface receptor, which consists of two EPOR molecules [80]. In non-neuronal cells, in a similar but more complex manner than in the hematopoietic system, EPO induces tyrosine phosphorylation of the EPOR and its associated kinase, Janus kinase 2 (JAK2); in fact, a comparable system has been addressed in neurons [81]. Endogenous and exogenous EPO can bind and stimulate the EPOR to induce phosphorylation of JAK2 [82,83]. Different receptors are involved in each tissue type and multiple neuroprotective signaling pathways are activated downstream of EPOR/JAK2 in the nervous system. Activated JAK2 induces various signaling pathways via several adaptor proteins such as phosphoinositide 3-kinase (PI3K), signal transducer and activator of transcription 5 (STAT5), nuclear factor kappa B (NF-κB) [84,85,86] and p42/44 mitogen-activated protein kinase (MAPK) [87]. All of these signaling pathways are known for promoting not only red blood cell proliferation, but also vasodilation [88], insulin-sensitization [89], and for having antithrombotic [90], anti-inflammatory and anti-apoptotic actions [91,92]. In particular, STAT5 and NFκB translocate into the nucleus and serve as transcription factors for Bcl-2 [93] and Bcl-xL [94], which are antiapoptotic genes. Both components of the signal transduction pathways (e.g., Akt/PKB) and gene products regulated by activated transcription factors (e.g., Bcl-2 and BclX) have been demonstrated to interfere with apoptotic processes in the nervous system [95].

The occurrence of EPOR splice variants [96] and the possible involvement of the EPOR-βcR heterodimer have received limited consideration [97]. Whereas homodimeric EPORs have been extensively studied, the existence of the heterodimeric complex is controversial and requires further study. Interestingly, similar signal transduction events, including activation of STAT5, are mediated via the EPOR-βcR hetero-receptor complex, which requires high local concentrations of EPO to be activated [98].

### 3.1. JAK2

JAK2 is a non-receptor tyrosine kinase involved in receptor signaling and hematopoiesis [99]. Both hematopoietic and non-hematopoietic effects are initiated by two tyrosine kinases of the JAK2 type, leading to trans- and EPOR-phosphorylation after receptor activation [100,101]. All dominant signaling pathways activated by EPO in erythropoiesis have also been implicated in the regulation of gene expression leading to neuroprotection [27]. Activated JAK2 induces a variety of signaling pathways that are known to affect the gene transcription involved in neuronal survival related to EPO [102,103].

### 3.2. STAT5

JAK2-mediated EPOR phosphorylation typically enables phosphorylation and dimerization of STAT transcription factors including STAT1, STAT3, and STAT5a/b [104,105], which translocate to the nucleus and activate regulated genes. These gene products can then interfere with apoptotic processes [56,106]. The family of mammalian STAT transcription factors regulates diverse functions implicated in developmental and homeostatic processes including apoptosis, growth, migration, proliferation, and differentiation [107,108].

In particular, STAT5, which mediates cellular responses to cytokines, growth factors, and hormones [109], has been implicated in EPO-stimulated erythropoiesis as well as protective mechanisms in various non-hematopoietic mammalian tissues including the nervous system [110]. It has been implicated in the control of neuronal cell fate decisions such as differentiation, proliferation, and apoptosis. Notably, EPO-mediated activation of JAK2/STAT5 leads to up-regulation of the anti-apoptotic *Bcl-XL* and *Bcl-2* genes, thereby protecting proerythroblasts from apoptosis [111,112].

### 3.3. NF-κB

NF-κB consists of homo- and hetero-dimers of five members of the Rel family: NF-κB1 (p50/p105), NF-κB2 (p52/p100), RelA (p65), RelB (I-REL), and c-Rel [113]. NF-κB can be activated by the phosphorylation of a tyrosine residue of the p50 subunit which then translocates into the nucleus after the release of IκB, and is known to be crucial factor in the differentiation of neuronal cells [114,115]. As NF-κB is known to be a downstream regulator of tumor necrosis factor (TNF)-α, it is particularly important in the neuroinflammatory processes involved in depression [116]. Given its major role in mediating inflammatory processes, many researchers have suggested that NF-κB is not only present in various immune cells but also on the surface of the BBB [117]. In the brain, proinflammatory cytokines activate both neuronal and non-neuronal cells (e.g., microglia, astrocytes, and oligodendroglia) via the NF-κB cascade in a similar manner to that occurring in the peripheral inflammatory response [118]. NF-κB activation is regulated by glucocorticoids which inhibit NF-κB activity, and decreases the activation of some pro-inflammatory cytokines in turn [119]. NF-κB is crucial for mediating the stress-induced inhibition of neurogenesis and at least some depressive behavior [120]. EPO-related production of forebrain neural stem cells (NSCs) [121] and prevention of neuronal apoptosis [122,123] require activation of NF-κB.

### 3.4. PI3K/Akt

PI3K/Akt signaling has been identified as the major transduction pathway for EPO-mediated cell protection in various mammalian non-hematopoietic tissues [124,125]. Previous studies have used the PI3K inhibitor LY294002 to prevent Akt phosphorylation and abrogate the protective effects of EPO [126,127,128]. The PI3K/Akt signal transduction pathway is known to play an important role in regulating major cellular processes, such as cell growth [129], proliferation and survival [130], cell metabolism, and autophagy [131]. There is also evidence that EPO can promote axonal growth and branching via activation of the PI3K/Akt pathway in polarized hippocampal neurons [132,133]. However other scientists who have studied mammalian neuroprotective and neuroregenerative signal transduction pathways for their contribution to rhEPO-mediated neuroprotection in locust brain neurons have demonstrated an involvement of JAK and STAT, but not of PI3K, in beneficial mechanisms that interfere with apoptotic processes [110]. This finding suggests that the pathways affected by EPO and its derivatives may be slightly different across species.

EPO markedly enhances the oxidative stress-sensitive activity of Akt and prevents the activation of microglia, which was one of the most important cellular components of neuroinflammation [134,135,136]. Since the inhibition of Akt phosphorylation blocks the cellular protective effects induced by EPO, Akt activity appears to be vital for EPO-mediated tissue protection [137]. Akt can also inactivate Bad, a member of pro-apoptotic Bcl-2 family, through phosphorylation of its serine residues [138]. EPO is linked to Bad through the anti-apoptotic Bcl-2 family member Bcl-xL. Studies have suggested that EPO was able to prevent cellular injury through maintaining the expression of Bcl-2 and Bcl-xL and altering the Bcl:Bax ratio [139]. EPO-induced activation of Akt also activated by phosphorylation of endothelial nitric oxide synthase (eNOS) [140]. Interestingly, Akt can significantly increase NF-κB and HIF-1 activation, resulting in increased EPO expression [141].

### 3.5. ERK/MAPK

The mitogen-activated proteins kinases (MAPKs) are a family of evolutionarily conserved molecules which play crucial roles in cellular signaling pathways and gene expression, consisting of three major members: Extracellular signal-regulated kinase (ERK), p38, and c-Jun N-terminal kinase [142], which represent three different intracellular-signaling cascades. Phosphorylation activates MAPKs, which transduce a broad range of extracellular stimuli into various intracellular responses through both transcriptional and non-transcriptional regulation [143]. Initiation of the ERK/MAPK cascade involves activation of three kinases, Ras, Raf, and MAPK/ERK kinase [144], and the ERK/MAPK pathway is customarily thought to play important roles in cell proliferation and differentiation [145]. Long-lasting activation of MAPK activity is known as a key mediator of cell differentiation [146], invariably involving translocation of ERK from the cytoplasm to the nucleus [147].

It is interesting to note that the MAPK family is involved in differentiation of neuronal cells [148,149] and astrocytes [150], and has been indicated to produce EPO, stimulating neuron or oligodendrocyte differentiation and accelerating the proliferation of astrocytes. MAPKs enter the nucleus and induce transcription of target genes involved mainly with inhibition of apoptosis and cell proliferation. In contrast with erythroid cell types, the EPO-mediated signaling pathways are less well characterized for non-erythroid tissues and, therefore, limited information on the mechanisms underlying the EPO-induced antidepressant effects is available.

## 4. EPO in the Treatment of Depression

The relationship of EPO and depression has been investigated in a number of studies. According to a systematic review, beneficial effects of EPO on hippocampus-dependent memory function and on depression-relevant behavior were observed in some animal and clinical studies, thus highlighting EPO as a candidate agent to manage cognitive dysfunction and mood symptoms in the future [151]. In some animal studies, EPO treatment can have some antidepressant effects, discriminable both morphologically and behaviorally [152]. Various behavioral tests, such as the forced swim test, novelty-induced hypophagia (NIH) test and novel object recognition test, proved useful in demonstrating improved cognitive function in rodent models (rat and mouse) following treatment with EPO [153]. Another study indicated that there was no effect on memory and depression- or anxiety-like behaviors three days after a single administration of EPO, but there was improvement of spatial and object recognition memory [154]. In additive, EPO in the brain can be induced by electroconvulsive seizures (ECS) and independently exhibits antidepressant-like effects according to the forced swim and NIH tests. Finally, analysis for gene expression profiles revealed that EPO alters the expression of neurotrophic genes such as BDNF [155,156].

In humans, beneficial effects of EPO on cognitive functions have been recognized as early as around the time of introduction of EPO for the clinical treatment of renal anemia [157,158,159]. In healthy volunteers, a single high dose of EPO reduced the neuronal response to fear one week after administration without evoking any erythropoietic alterations [160]. Following EPO administration, along with increased neural and cognitive processing of facial expressions, a short-term effect of improved mood symptoms was reported in the first three days, which is similar to the neuro-behavioral effects obtained in acute administration of selective serotonin reuptake inhibitor (SSRI) antidepressants [161]. Three days post-administration of EPO also showed decreased neural responses to negative *vs.* positive pictures in a network of sites including the hippocampus [162]. However, another study showed that ARA290, an EPO-derived peptide, tended to lower the recognition of facial expressions of happiness and disgust and had no effects on mood and affective symptoms [163]. A double-blind study comprising 19 patients with acute depression provided evidence that EPO was found to reduce left amygdala-hippocampal response to fearful stimuli [164]. Some randomized controlled studies indicated EPO may provide a therapeutic option for patients with mood disorders [164,165,166,167]. Recently, clinical evidence of the procognitive potential of EPO has been proved by a randomized controlled trial (RCT) involving moderately depressed patients with treatment-resistant depression (*N* = 40) [168]. EPO also down-regulated plasma BDNF levels in patients with treatment-resistant depression (TRD; *N* = 40) [169]. Taken together, these seven clinical findings (Table 1) suggested that EPO may provide a therapeutic option for patients with depression. Nonetheless, all of these clinical studies have some limitations, such as the small sample sizes of patients, concurrent use of antidepressant medications in many patients, incomplete examination of mood and arousal changes, and unclear baseline data.

The mechanism of depression is very complex. A shrinkage of the hippocampal volume in depressive patients [170] and a decreased number of astrocytes and neurons in the prefrontal cortex and striatum [171] associated with depression were observed in some clinical and experimental studies. Scientists have examined and advanced the theory that depression is an inflammatory disorder [172,173] and is related to neuroprogression, especially in the hippocampus [174]. Indeed, one hypothesis that has recently gained traction suggests that depression is caused by a breakdown in neural plasticity arising from on-going inflammatory processes and an overactive stress-response system [175,176], which leads to structural and functional abnormalities in the fronto-limbic brain circuitry [177]. Based on the pharmacological effects of EPO in the nervous system, it might, for example, attenuate neuroinflammatory processes [178], and improve hippocampal neurogenesis [179]. Consistent with the above theory of depression, there is reason to believe that EPO pathways could be a potential target for the treatment of depression.

### 4.1. Inflammation in EPO-Related Treatments

A recent meta-analysis study indicated that depression is characterized by increased levels of IL-6 and TNF-α in plasma, which is interpreted to indicate that depression may be considered as an inflammatory disorder [180]. It was also shown that systems related to the mitigation of the inflammatory response may be potential therapeutic targets for mood disorders [181]. Non-steroidal anti-inflammatory drugs (NSAIDs), such as acetylsalicylic acid and celecoxib, have an adjunctive effect when combined with SSRIs in the treatment of clinical and experimental depression [182].

EPO is considered to have potential anti-inflammatory capacities, especially as evidenced by its successful application in a number of animal models of chronic inflammation [183,184]. EPO impairs the formation of pro-inflammatory factors such as TNF-α, IL-6, IL-12/IL-23 subunits and nitric oxide (NO) via induction of inducible NO synthase (iNOS) in macrophages [185]. It also has anti-inflammatory effects by reducing reactive astrocytosis and microglia activation and the number of immune cells in the injured sites [186,187]. The mechanisms of these anti-inflammatory effects have not been investigated widely; however, a recent report showed that inhibition of the NF-κB p65 subunit is likely to be essential [188].

### 4.2. Neuroprogression in EPO-Related Treatment

Neuroprogression is defined as the progression of neurodegeneration, apoptosis, and reduced neurogenesis, and structural, functional, molecular, and cellular modifications and neuronal plasticity; together, these phenomena most likely result from inflammation and other factors [189]. Depressive disorder is related to some structural brain changes, such as decreased hippocampal volume [190], which might come from a stressors-caused decrease in neurogenesis [191,192].

EPO has been implicated in the accommodation of neuroprogression and may play an important antidepressant role in the progress of depression. According to some experiments, EPO improved antidepressant and anti-anxiety-like effects in the forced swim test, which related to significantly increased hippocampal neurogenesis [193]; however, no evidence of a general EPO-related increase in mobility was observed in the open field test [155]. Systemically administered EPO crosses the BBB in therapeutically effective concentrations [194] and exerts neuroprotective and neurotrophic effects [195] in traumatic, hypoxic-ischemic, excitotoxic, and inflammatory brain damage [196,197], and in neurodegenerative and neuropsychiatric conditions [198,199,200]. These morphological effects of EPO are caused by direct action on neurons through EPO-EPOR pathways and are strongly correlated with brain-derived neurotrophic factor (BDNF), which plays a crucial role in neuronal survival and proliferation [201]. BDNF and EPO share a common set of intracellular signaling pathways including the PI3K and MAPK cascades [202,203,204]. EPO was reported to induce BDNF expression, inducing potential neuroplastic effects [154].

## 5. Conclusions and Perspective

Depression is a global issue and the leading cause of burden and disability worldwide. This very complex psychosocial and biological phenomenon contains intricate neurophysiological, behavioral, psychosocial, and affective constituents. The underlying mechanisms of depression have been difficult to illuminate because of the heterogeneous nature and the different etiologies of the disease. One potentially valuable theory states that not only the alterations in the volume of the hippocampus, prefrontal cortex, thalamus, and basal ganglia, but also inflammatory conditions are related to the causative mechanisms of depression. These findings suggest that targeting several pathophysiologic mechanisms rather than neurotransmitter systems specifically holds promise for developing innovative therapeutic strategies.

During the past decade, accumulating evidence has indicated that EPO may have potential as a treatment for depression, suggesting that endogenous cytokines may play an important role in the pathogenesis of depression. In addition to the kidney, liver, and uterus, many organs have been identified as secretory tissues for EPO, including the brain. The EPOR is expressed in cerebral and spinal cord neurons, and in the hypothalamus, hippocampus, neocortex, dorsal root ganglia and nerve axons. The discovery of EPO and EPOR in the nervous system highlights new functions for EPO, which was only considered to play a crucial role in the progress of erythroid differentiation. Mounting experimental evidence suggests that EPO treatment, which has clearly shown antidepressant and procognitive effects, may alleviate inflammation and neuroprogression in depression models. Therefore, exploring EPO-EPOR and their downstream signaling pathways may greatly improve our understanding of the pathogenic mechanisms that underlie depression and the molecular basis of its treatment. EPO-EPOR signaling through JAK2 activates multiple downstream signaling pathways including STAT5, PI3K/Akt, NF-κB, and MAPK. These factors may play an important role in inflammation and neuroprogression in the nervous system, particularly in the hippocampus, which is heavily involved in the development of depression. Unfortunately, to date there have been few reports about the relationship between EPO and depression. Thus, the role of the EPO-EPOR pathway in the treatment of depression makes it an attractive target for the development of new approaches to treating depression. This will also help to identify new targets for pharmacological intervention.

However, with the numerous effects attributed to EPO, several aspects of potential EPO treatment for depression must be clarified.

First, in the coming years, it will be crucial to evaluate the level of the expression of EPO or EPOR and to inhibit their downstream effectors to unequivocally define the role of EPO-EPOR pathways in depression processes. EPO antibodies and EPOR antagonists should be used in studies of the relationship between depression and EPO-EPOR pathways. Further research is necessary to determine the exact role of EPO-EPOR pathways in the progress of depression, including activation of EPO and specific subtypes of its signal transduction. In fact, this will likely be an active area of research for many years to come. As has been reported, derivatives such as carbamylated erythropoietin (CEPO) and asialo-EPO may also have neuroprotective functions. These derivatives need to be examined in greater detail. Development of new therapeutics to treat depression provides significant evidence of our new understanding of its homeostasis and pathophysiological features.

Second, EPOR is widely expressed in several tissues, including the muscle, liver, heart, and spinal cord, where it might be involved in physiological and pathophysiological processes, including tissue protection and immunomodulation. As we know, depression is associated with many neurological disorders and other chronic physical health conditions, such as diabetes, chronic liver disease, heart disease, and cardiovascular disease. It is, therefore, possible that studying EPO-EPOR pathways will help to explain the connection between depression and other disabilities.

Third, it is unclear whether the EPO-triggered signaling cascades differ across tissue, whether one cell type expresses both types of EPORs, and how these types of EPORs might differentially affect EPO-induced cellular and intracellular pathways and effects. Whether some other cytokines such as interleukin-3 (IL-3), granulocyte-macrophage colony-stimulating factor (GM-CSF) or IL-5 can signal through the EPOR-βcR complex, interfere with, or modify EPO signaling pathways remains to be unknown. In the future, animal models, such as gene knock-in and knock-outs of EPOR or βcR should be developed to study these issues.

Finally, despite the promising evidence for EPO as an additional treatment for mood disorders, it is important to acknowledge one major limitation of EPO. The hematopoietic action of EPO with repeated administration would necessitate close monitoring of hematocrit and thrombocyte levels and, potentially, blood-letting in non-anemic patient populations. Some studies show that exogenous EPO-treated cancer patients have been associated with tumor progression [205,206,207,208], which suggests the potential risk of the use of EPO. However, with further research discerning between the mechanisms of its antidepressant and hematopoietic effects, we may be able to develop EPO derivatives with antidepressant effects that lack a hematopoietic function.

In conclusion, research to identify compounds and therapeutic strategies targeting EPO pathways may be essential for the treatment of depression. Furthermore, extensive clinical trials are required to evaluate more effective and safer drugs related to EPO pathways in humans.

## Figures and Tables

**Table 1:** Clinical study on the antidepressant effect of EPO.

Author	EPO Form	Subject	Drug Administration	Randomized	Double-Blind	Main Finding	Limitations	Safety
Kamilla W. Miskowiak *et al.* (2008) [161]	Erythropoietin (40,000 International Unit, IU)	healthy volunteers (*N* = 24)	injection once	Y	Y	During faces processing EPO enhanced activation in the left amygdala and right precuneus to happy and fearful expressions. This was paired with improved recognition of all facial expressions, in particular of low intensity happiness and fear. This is similar to behavioral effects observed with acute administration of serotonergic antidepressants.	1, pharmacological fMRI studies in general is the possibility that drug effects on neural response may be confounded by non-specific effects on neural coupling and cerebral hemodynamics. 2, more detailed examination of the mood and arousal changes seen following EPO and their relation to changes in emotional processing observed three days post-administration should be performed. 3, the clinical effect in patients suffering from depression is unknown.	Blood pressure and subjective state were monitored for 2 h following the injection.
Hilâl Cerit *et al.* (2008) [163]	ARA290 (2 mg)	healthy volunteers (*N* = 36)	injection, once	Y	Y	ARA290-treated individuals displayed lower neural responses to happy faces in the fusiform gyrus. ARA290 tended to lower the recognition of happy and disgust facial expressions. Although ARA290 was not associated with a better memory for positive words, it was associated with faster categorization of positive *vs.* negative words. Finally, ARA290 increased attention towards positive emotional pictures. No effects were observed on mood and affective symptoms.	1, the limited clinical potential of EPO to treat depressive symptoms in non-anemic patients, due to the hematopoietic actions of EPO with repeated administration. 2, the human proof-of-concept studies were conducted in relatively small samples.	After administration, the participant was monitored for 10 min. Dose selection was based on previous studies in humans in which no safety concerns were reported.
Kamilla W. Miskowiak *et al.* (2009) [162]	Erythropoietin (40,000 IU)	depressed patients (*N* = 17)	injection once	Y	Y	EPO reduced neural response to negative *vs.* positive pictures three days post-administration in a network of areas including the hippocampus, ventromedial prefrontal and parietal cortex. After the scan, EPO-treated patients showed improved memory compared with those that were given placebo. The effects occurred in the absence of changes in mood or hematological parameters, suggesting that they originated from direct neurobiological actions of EPO.	1, an exploratory study in a small patient sample. 2, The majority of patientswere also taking antidepressant medication	Blood pressure, well-being and subjective state was monitored for 2 h following the injection.
Kamilla W. Miskowiak *et al.* (2010) [164]	Erythropoietin(40,000 IU)	depressed patients (*N* = 19)	injection once	Y	Y	EPO reduced neural response to fearful *vs.* happy faces in the amygdala and hippocampus, and to fearful faces *vs.* baseline in superior temporal and occipitoparietal regions three days after administration in acutely depressed patients. This was accompanied by a specific reduction in the recognition of fear in EPO-treated patients after the scan similar to the effects on face recognition seen with antidepressant drug treatment.	1, an exploratory study in a small patient sample. 2, the majority of patients were taking antidepressant medication. 3, the current study used a between-groups design, and it is unknown whether baseline differences existed between the two groups. 4, the application of EPO in the treatment of neuropsychiatric disorder may have some undesirable effects.	Following EPO/saline administration, blood pressure, well-being, and subjective state were monitored for 2 h.
Kamilla W. Miskowiak *et al.* (2014) [168]	Erythropoietin(40,000 IU)	depressed patients (*N* = 40)	injection weekly (8 weeks)	Y	Y	HDRS-17, GAF, and remission rates showed no effects of EPO over saline at week 9. However, EPO improved BDI and WHOQOL-BREF, and this was maintained at follow-up week 14. EPO enhanced verbal recall and recognition, which was sustained at follow-up. Exploratory analysis in patients fulfilling depression severity criteria at trial start revealed ameliorated HDRS-17 in EPO *vs.* saline groups, which was sustained at week 14. Exploratory analysis in the complete cohort showed that EPO reduced depression composite at weeks 9 and 14.	1, the extensive exclusion criteria may limit the ability to generalize our findings to clinical practice. 2, the EPO-associated increase in red blood cell levels could confound the interpretation of the effects of EPO as neural in origin. 3, they did not screen for or exclude co-morbid axis II disorder as this would have resulted in a subsample of patients who were not representative for the target population of treatment-resistant patients. 4, their study may not have been adequately powered to detect a significant effect on primary outcome measure, although a positive signal was apparent on the additional depression-relevant outcomes and explorative score of depressive syndrome severity. 5, patients had been treated for years without any improvement, and that a treatment period of eight weeks is very short in such chronic, recurrent condition.	Weekly monitoring of blood tests and any side effects was performed by a physician not involved in outcome measure assessments.
Kamilla W. Miskowiak *et al.* (2015) [166]	Erythropoietin(40,000 IU)	BD/TRD patients (*N* = 69 )	injection weekly (8 weeks)	Y	Y	Compared with saline, EPO was associated with mood-independent memory improvement and reversal of brain matter loss in the left hippocampalcornu ammonis 1 to cornu ammonis 3 and subiculum. Using the entire sample, memory improvement was associated with subfield hippocampal volume increase independent of mood change.	1, their cohort included both patients with TRD and BD, since these mood disorders may involve differential, although partially overlapping, pathogenic processes. 2, three complementary methods to capture different aspects of hippocampal volume changes have their own limitations, and reflect different structural measures.	Blood tests were taken on a weekly basis from baseline to week 10 (two weeks after treatment completion) and again in week 14.
Maj Vinberg *et al.* (2015) [169]	Erythropoietin(40,000 IU)	BD/TRD patients (*N* = 83 )	injection weekly (8 weeks)	Y	Y	EPO down-regulated plasma BDNF levels in patients with TRD (mean reduction at week 9 (95% CI): EPO 10.94 ng/L (4.51–21.41 ng/L); mean increase at week 9: Saline 0.52 ng/L, *p* = 0.04 (−5.88–4.48 ng/L) *p* = 0.04, partial η2 = 0.12). No significant effects were found on BDNF levels in partially remitted patients with BD (*p* = 0.35).	1, they did not register daily physical exercise level, and since EPO is well known for its potential doping capacity, the change in BDNF levels could be due to increased exercise levels in the intervention group. 2, the relatively few participants is a limitation. 3, patients received weekly intravenous infusions of either EPO or saline for eight weeks (weeks 1–8) in addition to their current antidepressant medication.	Blood tests were taken on a weekly basis from baseline to week 10.

## Abbreviations

EPO	Erythropoietin
EPOR	Erythropoietin receptor
βcR/CD131	β-common receptor
5-HT	5-hydroxytryptamine
BDNF	Brain-derived neutrophic factor
ECs	Endothelial cells
BBB	Blood brain barrier
GM-CSF	granulocyte-macrophage colony-stimulating factor
IL-3	Interleukin 3
IL-5	Interleukin 5
EphB4	Ephrin-type B receptor 4
RhEPO	Recombinant human EPO
CEPO	Carbamylated erythropoietin
HIF	Hypoxia-inducible factor
ROS	Reactive oxygen species
JAK2	Janus kinase 2
PI3K	Phosphoinositide 3-kinase
NF-κB	Nuclear factor kappa B
STAT5	Transducer and activator of transcription 5
Akt	Protein Ser/Thr kinase
MAPK	P42/44 mitogen-activated protein kinase
SSRI	Selective serotonin reuptake inhibitor
NSAIDs	Non-steroidal anti-inflammatory drugs
NO	Nitric oxide
iNOS	Inducible NO synthase
